# Transmission of the frequency components of the vibrational signal of the glassy-winged sharpshooter, *Homalodisca vitripennis,* within and between grapevines

**DOI:** 10.1007/s00359-019-01366-w

**Published:** 2019-08-23

**Authors:** Shira D. Gordon, Benjamin Tiller, James F. C. Windmill, Rodrigo Krugner, Peter M. Narins

**Affiliations:** 1 Agricultural Research Service, San Joaquin Valley Agricultural Sciences Center, United States Department of Agriculture, 9611 S Riverbend Ave, Parlier, CA 93648 USA; 2 Department of Electronic and Electrical Engineering, Centre for Ultrasonic Engineering, University of Strathclyde, Glasgow, Scotland G1 1XW UK; 3 Department of Integrative Biology and Physiology, UCLA, Los Angeles, CA 90095 USA

**Keywords:** Vibrational communication, Signal transmission, Bending waves, Active space, Dispersion

## Abstract

The agricultural pest, *Homalodisca vitripennis*, relies on vibrational communication through plants for species identification, location, and courtship. Their vibrational signal exhibits a dominant frequency between 80 and 120 Hz, with higher frequency, lower intensity harmonics occurring approximately every 100 Hz. However, previous research revealed that not all harmonics are recorded in every signal. Therefore, how the female *H. vitripennis* vibrational signal changes as it travels through the plant was investigated. Results confirmed that transmission was a bending wave, with decreased signal intensity for increasing distance from the source; moreover, at distances of 50 cm, higher frequencies traveled faster than lower frequencies, suggesting that dispersion of *H. vitripennis* signal components may enable signaling partners to encode distance. Finally, *H. vitripennis* generates no detectable airborne signal (pressure wave), yet their low vibrational frequency components are detectable in neighboring plants as a result of leaf-to-air-to-leaf propagation. For instance, with isolated key female signal frequencies, 100 Hz was detected at a 10 cm gap between leaves, whereas 600 Hz was detectable only with a 0.1 cm gap. Together, these results highlight the complexity of vibration propagation in plants and suggest the possibility of the animals using the harmonic content to determine distance to the signaling *H. vitripennis* source.

**Electronic supplementary material:**

The online version of this article (10.1007/s00359-019-01366-w) contains supplementary material, which is available to authorized users.

## Introduction

Signaling is rarely done in isolation, but must be evaluated in the context of the environment. The distance from the source, over which signal amplitude remains above the detection threshold of potential receivers (active space, Brenowitz 1982), plays a key role for both signal transmission and reception. During signaling, animals are often capable of adjusting their communication behaviors to maximize the reach of their efforts, in some cases using multimodal signals (Narins et al. 2003, 2005; Partan and Marler 2005), by adjusting signal intensity (Lopez et al. 1988; Egnor et al. 2007) or acoustic frequency (Lardner and bin Lakim 2002), or by emphasizing certain components of the signal (Wells and Schwartz 1984; Halfwerk and Slabbekoorn 2009; Gordon and Uetz 2011). However, the signaling active space must be considered. For example, amplitude alone may not provide enough information to a plantborne receiver; thus, both the amplitude and frequency components of the vibrational signal must be considered within the plant (Mazzoni et al. 2014). From the receiver’s perspective, the signal itself may give important contextual cues about the signaler’s distance, location, or quality.

Many arthropod species rely primarily on vibrations for communication and signaling (Barth 1997, 1998; Rovner and Barth 1981; Michelsen et al. 1982; Cocroft and Rodriguez 2005). These vibrational patterns provide the *who*—species, *what*—attractive partner, *where*—directional cues, and *when*—timing for a duet. The properties of the plant substrates may alter the transmitted signal (e.g., frequency filtering, attenuation, and dispersion) (Mortimer 2017). Small changes in vibrational signal components such as timing and frequency may affect communication success (Čokl et al. 2015). While the dominant frequency is usually a key component in signal recognition, there are often multiple harmonics present in vibrational signals (Čokl and Virant-Doberlet 2003). Although some studies have investigated the frequency dependence of an insect’s response, less is known about the nature of the information present in the harmonics.

Vibrational signaling on many plant species often may be reduced to the problem of a bending wave traveling in a beam (Markl 1983; Miles 2016). As such, signals under 5 kHz are dispersive, meaning the velocity of propagation is frequency dependent (Markl 1983; Casas et al. 2007). Furthermore, wave reflections from the branch ends interfere with oncoming waves to produce standing waves, i.e., local regions of low and high vibration amplitudes along the branch (nodes and antinodes); the locations of these are wavelength and, therefore, frequency dependent (Polajnar et al. 2012). Reflections not only from branch ends, but also from plant stems, branch nodes, and leaves may also alter the original vibrational signal (Michelsen et al. 1982; Čokl and Virant-Doberlet 2003). In a dispersive system, the vibration amplitude of the signal will decrease and the overall signal will have fewer high-frequency components with increasing distance from the source, but what does this mean for the animals? How, if at all, is this differential attenuation of high-frequency components and harmonics received by a distant receiver?

In a related question, recent work shows that vibrational signals may propagate between two leaves of adjacent but non-connected plants (Eriksson et al. 2011). Behavioral studies determined that 80% of leafhoppers, *Scaphoideus titanus,* engage in vibratory duets with an air gap of 5 cm between two leaves occupied by individual animals (Eriksson et al. 2011). That study included a general frequency–velocity analysis identifying a decrease in intensity and an increase in the dominant frequency across the gap between plants. However, a specific frequency analysis of the signal was not a part of the scope of that study. Therefore, a more specific analysis of individual frequency transmission across the gap between the sending and receiving leaves may be informative.

**Fig. 1:**
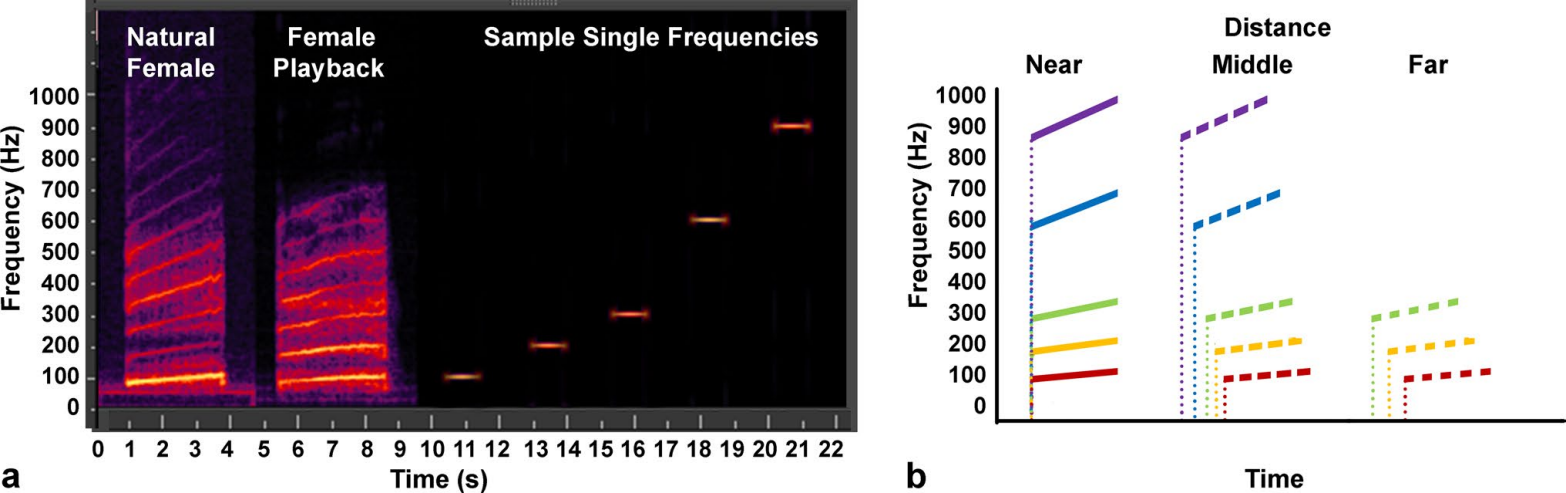
**a** Sample of a call from a female *Homalodisca vitripennis*, playback of a female signal, and individual frequencies. Color spectrum indicates intensity with yellow and black representing the highest and lowest signal intensities, respectively. **b** Model of signal dispersion and attenuation at different distances over time

The goal of the present study was to investigate signal transmission within and between the plants. The focus was the vibrational signal of female glassy-winged sharpshooter, *Homalodisca vitripennis* (Germar). *H. vitripennis* was chosen because it is a common grapevine pest in California, transmitting a bacterium, *Xylella fastidiosa*, which can kill the grapevine in 2−3 years post-infection. Recent research has characterized *H. vitripennis* signals (Nieri et al. 2017) and has shown that playback of female signals is sufficient to disrupt mating both in the laboratory (Gordon et al. 2017) and in the field (Krugner and Gordon 2018). Methods were established to match the amplitude of natural signals with those of synthetic signals used in playback experiments (Krugner and Gordon 2018; Fig. 1a). In addition, studies showed that deletion of high-frequency harmonics of a female signal does not affect male signaling response (Mazzoni et al. 2017), which demonstrates the relative importance of the dominant frequency and the first two harmonics (i.e., 100, 200, 300 Hz). The female signal has a duration of 1−4 s, a dominant frequency that sweeps upward from approximately 80−120 Hz, and consists of additional harmonics approximately every 100 Hz up to at least 1 kHz (Fig. 1a). To obtain consistent playback signals, we used computer-generated sweeps emulating the female signal components (Fig. 1a). We measured signal transmission within a 50 cm section along a grapevine as well as between two adjacent but non-connected leaves from different plants. We analyzed the intensity, time of arrival, latency, and amplitude of the transmitted frequency components to determine the specifics of *H. vitripennis* signal transmission.

While previous research has investigated the transmission velocity of vibrational signals in plants (Casas et al. 2007; Miles 2016; Polajnar et al. 2012), here we considered what this information may actually mean during transmission, and therefore, to the receiving insect. *H. vitripennis* is an ideal system, as the female signal is relatively simple (Fig. 1a) and stimulates the important male searching behaviors. After the initial 1:1 signaling pattern between the male and female (duets), the male begins to search for the female on the plant by leading the duet with or without a female response. During this phase, the specific cues used by the male to find the female and what stimulates the female to sporadically respond are unknown. Therefore, in this study, we tested the feasibility of the idea of using the higher frequencies as a signaling component as a result of dispersion, or frequency-dependent propagation velocity. We hypothesized that if the grapevine is truly a dispersive system, then for short distances, the signal components should arrive nearly simultaneously with approximately their original source amplitudes (Fig. 1b). However, as the distance from the source increases, the high-frequency signal components will arrive earlier than the low-frequency signal components, thus altering the signal waveform perceived by the receiver. Likewise, as the signal propagates, attenuation will differentially affect the signal components (Fig. 1b), resulting in further deterioration of the original signal waveform. Thus, by evaluating the amplitudes of the individual frequency components (e.g., 100, 200, 300, 600, and 900 Hz) that compose the *H. vitripennis* signal, we evaluated if this proposed model of dispersive signal transmission is viable, and if so, its potential to provide additional source distance cues for the animal.

## Materials and methods

### Signals and playback

**Fig. 2:**
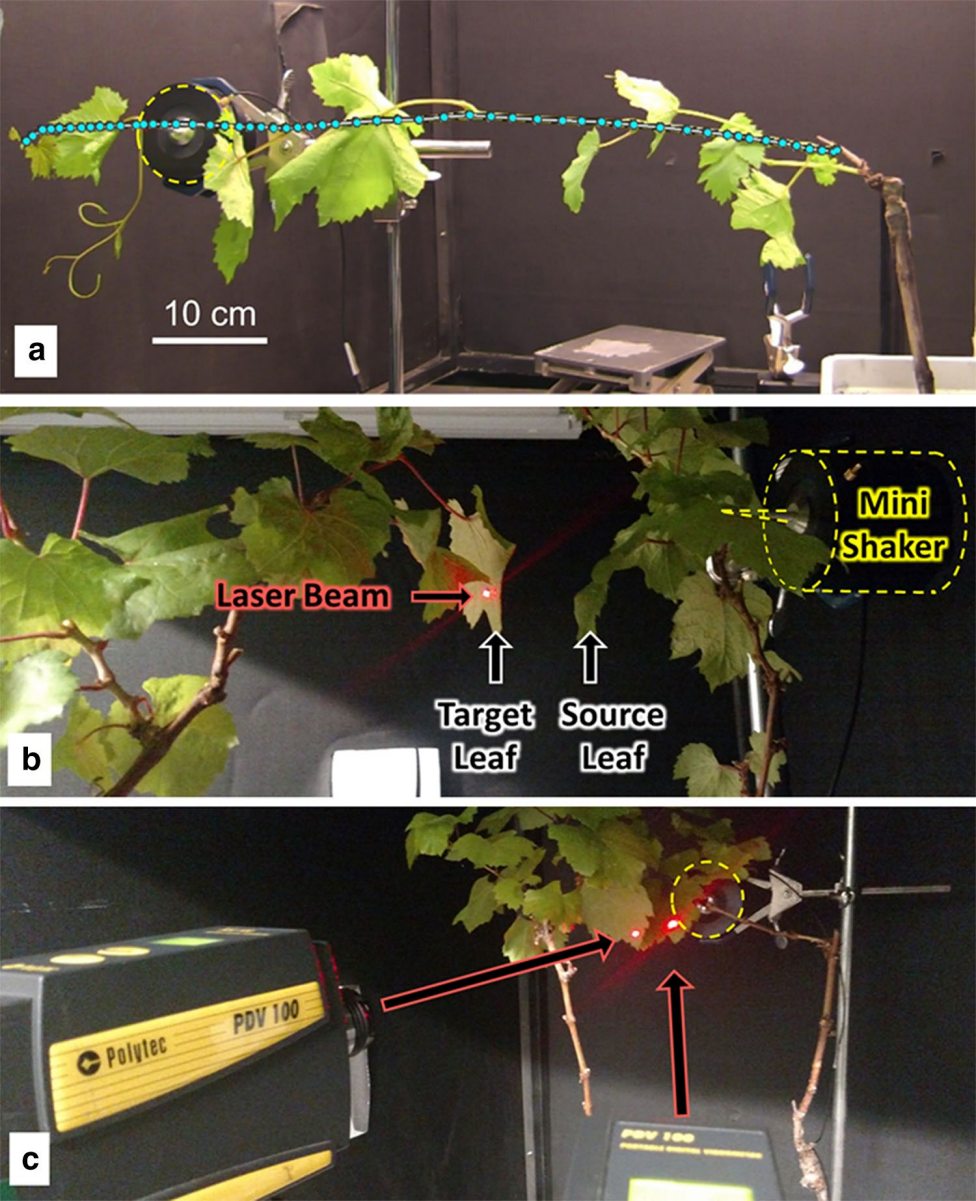
**a** Sample of the experimental setup. Light blue circles indicate the scanning laser vibrometer measurement points along the grapevine; **b**, **c** plant arrangement used to measure sound transmission from leaf-to-air-to-leaf seen from **b** perpendicular, and **c** in-line views. The mini-shaker is outlined with a dashed yellow line in each panel

Pure-tone signals were software generated (Polytec, PSV 9.3) as 0.025 s sweep tones over 40 Hz centered at 100 Hz, 200 Hz, 300 Hz, 600 Hz, and 900 Hz and were separated by silent periods of 0.025 s and 0.35 s preceding and following the signal, respectively. Vibrational stimuli for all experiments were delivered with a mini-shaker (type 4810, Brüel & Kjær, Denmark) with a custom stinger attached to the device (Fig. 2). The stinger consisted of a screw with the top end filled to a point that was inserted firmly into the plant. The plants used were potted grapevines (*Vitis vinifera* L. cv Chardonnay), with branches approximately 3−4 mm thick where measurements occurred. All experiments were conducted on a vibration isolation table (Model 20-561, Technical Manufacturing Corporation, Peabody, MA, USA).

### Recording signals through the branch

Vibration velocities on the plant were measured using a scanning laser Doppler vibrometer (SLDV, PSV-500 Polytec Inc., Irvine, CA, USA) at distances between 1 and 50 cm from the mini-shaker attachment point (Fig. 2a). The trigger signal was generated internally with averaging set to three scans per point. In addition, a second single-point laser Doppler vibrometer (PDV-100, Polytec Inc., Irvine, CA, USA) was aimed at the mini-shaker attachment point on the grapevine. Both vibrometer outputs and the trigger signal were recorded simultaneously on separate channels of a laptop computer.

### Analysis of scanning trials

**Fig. 3:**
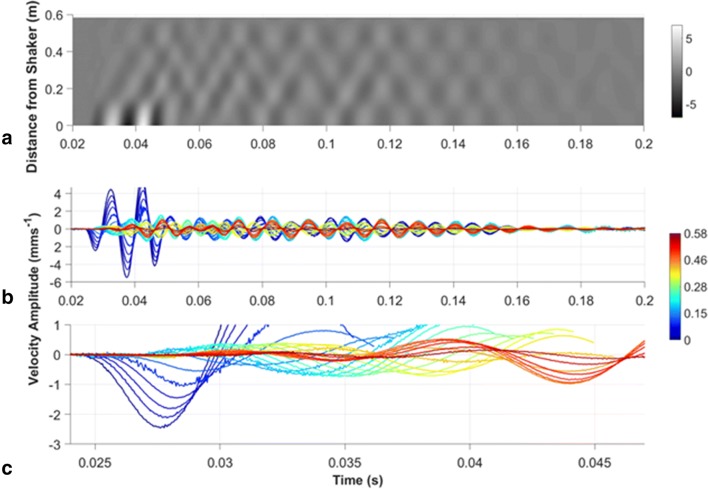
Sample data of signal transmission on one vine at 100 Hz in **a** matrix plot of time and distance (gray scale is velocity amplitude mm s^−1^); **b** time vs. velocity amplitude at different distances from the source (colors are the distances from the source in meters, with red at the mini-shaker to blue furthest away); **c** magnified view of **b** showing details of the low-amplitude traces. Sample data from different frequencies can also be seen as a video representation for each frequency in the supplemental materials (suppl. video 1−6)

For each measurement point along a branch, the position coordinates and the averaged time–velocity data were extracted by the Polytec software. The data points were then placed on a universal distance scale with 0 cm being at the mini-shaker attachment point. The distance traveled along the branch to each point was calculated by summing the distance between nearest neighboring points using the *x*-, *y*-, and *z*-coordinates from the scan. Using Matlab, a data matrix was then created for each vine and visualized as a surface plot, where the *x*-axis corresponded to time and the *y*-axis to distance traveled along the branch (e.g., see Fig. 3a and suppl. video 1). The color scale corresponds to velocity magnitude, allowing visualization of the movement of each phase front along the branch. From these plots, phase velocity was calculated by measuring the distance traveled by a crest or trough as it moved along the branch. Wavelength for each individual frequency was calculated by taking the phase velocity value and dividing by the frequency. Since the vibrometer output voltage is proportional to the target velocity (mm/s), these values translate to displacement by

1 $$d = \frac{v}{\omega },$$

where *d* is the instantaneous displacement, *v* the instantaneous velocity and $\omega = 2\pi f$ is the angular frequency.

Arrival time was taken for set distances every 10 cm by plotting the data in Excel and identifying the time point corresponding to the wave’s first peak. Because the signal started after the initial 0.025 s silent period described above, this time was subtracted from all reported values. To ensure the correct peak was measured, the peak of the first wave (see Fig. 3b, c) had to follow the previous time point and could not arrive before the time point of the previous distance. The absolute value of the maximum intensity was identified in Excel for each point.

Means and standard deviations were reported; sample size was eight plants. Wavelength, phase velocity, and intensity were analyzed with an one-way ANOVA in the statistical package JMP (SAS Institute Inc., Cary, NC, USA) to identify any significant changes with increasing frequency. A two-factor crossed ANOVA was used to determine the effect of frequency and distance on the arrival time of the wave.

### Leaf–air–leaf signal transmission

Two potted grapevines were used for the trials: one was designated as the source leaf and the other as the target leaf (Fig. 2b, c). The mini-shaker tip was attached to a point 2 cm from the source-leaf petiole. The target plant was initially positioned with the target leaf in direct contact with the source leaf. Both plants were placed on sound-dampening pads to attenuate substrate vibrational transmission between them; as an additional control, we measured the background vibrational levels without the source plant (see description below). Leaves were chosen to have relatively flat, vertical surfaces. Leaves (*n* = 16) were approximately 9 × 7 cm (w × l, ± 1.0 cm, 0.6 cm s.d.) equating to a mean area of 54 cm^2^ (± 8.8 cm^2^ s.d.) with a petiole length of 4 cm (± 0.8 cm s.d.). Leaf area was calculated immediately after the trials using ImageJ (Schneider et al. 2012). Plants were used once as a source and once as a target, but never with the same paired plant combination; ten pair combinations were used.

For each trial, the separation between the source plant leaf nearest to the target plant and the target plant leaf nearest to the source plant was systematically increased from 0 (touching) to 0.1 cm (not touching) to 1 cm and thereafter increased in 1 cm steps to 10 cm (Fig. 2b). Inter-plant distance was measured between the closest points of the leaves. Once the maximum distance was reached, the background vibration level was measured as a control. The source plant was removed leaving the shaker free in the air, not touching any plant and the target plant was incrementally moved back to its original location to acquire the background sound levels. Vibration measurements were recorded with two single-point vibrometers focused on a small square (5 mm^2^) of reflective tape placed on the leaves (Fig. 2c). When the source plant was removed, the source vibrometer was focused on the mini-shaker tip.

Signals were recorded and analyzed using Adobe Audition (Adobe Systems, San Jose, CA, USA). The entire recorded signal was highlighted and an FFT (No. of points: 4096; window: Hamming) was calculated. Signal intensities were measured as relative dB and compared to the background sound level, when no source plant was present. Using these methods, the distance at which the signal was no longer identifiable above the background noise was determined. Data were analyzed in a matched-pair design using the statistical software JMP by comparing the intensity of each point to the background intensity at the same distance (*n* = 10).

## Results

### Single-frequency transmission

**Fig. 4:**
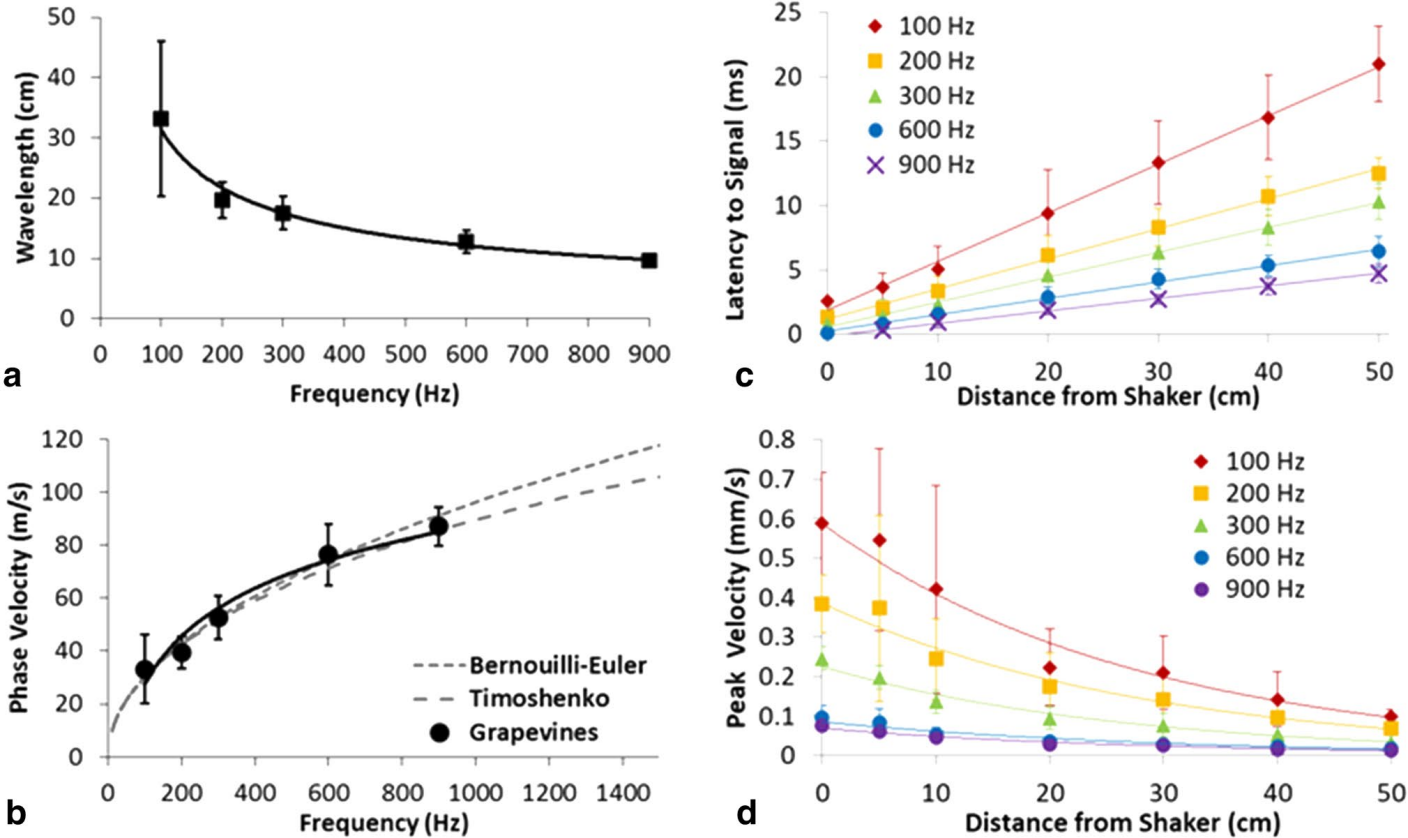
**a** Wavelength, **b** phase velocity, **c** latency, and **d** absolute value of the maximum intensity for signal arrival along the branch. All values are mean ± standard deviation; best fit are lines graphed with the data. In **b**, two-phase velocity theories of lower frequencies (< 5 kHz) traveling at different speeds in substrates are also graphed with the data

A sine wave was measured traveling along the branch, detectable at different distances (Fig. 3, Suppl Videos 1−6). With increasing frequency, the wavelength decreased (*F*_4,38_ = 75.67, *p* < 0.0001) and the wave speed increased (*F*_4,38_ = 74.48, *p* < 0.0001) (Fig. 4a, b). Therefore, the further away from the source, the longer the signal took to arrive in a frequency-dependent fashion (*F*_4,38_ = 94.13, *p* < 0.0001) (Fig. 4c). For example, at 50 cm from the source, the 600 Hz signal arrived 15 ms prior to the 100 Hz signal. As the distance increased, the amplitude of each frequency decreased (for all frequencies *p* < 0.0001; 100 Hz: *F* = 12.85; 200 Hz: *F* = 9.83; 300 Hz: *F* = 28.95; 600 Hz: *F* = 20.75; 900 Hz: *F* = 46.85) (Fig. 4d).

### Leaf-to-leaf transmission

**Fig. 5:**
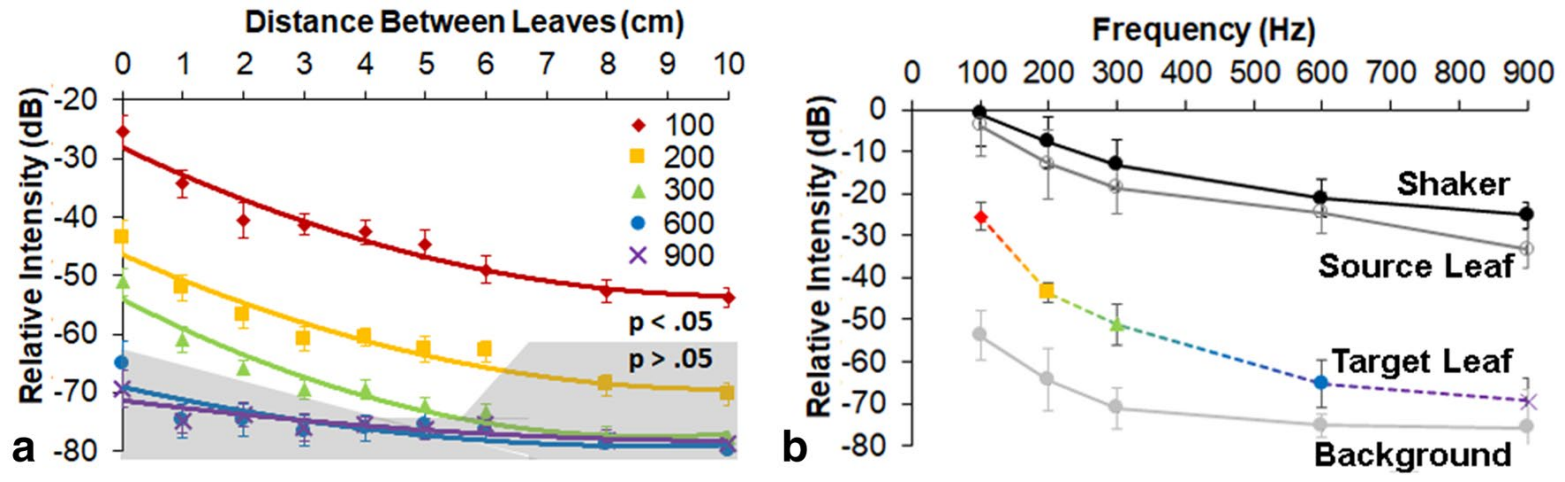
**a** Plot of the signal received on leaves from an adjacent, non-connected plant. Data are transmitted to the second plant (*p* < 0.05) except in the gray areas. **b** Signal intensity from the tip of the shaker, the source leaf, the target leaf (0.1 mm away), and the background noise (at the target leaf at 0.1 mm away, with no source plant)

The active space of signal transmission extended beyond touching plants (Fig. 5). At 100 Hz, the frequency with the longest wavelength tested, the signal was detectable above background noise at the furthest distance tested, 10 cm (10 cm: *t* ratio = 3.34, *p* = 0.009). Both 200 Hz and 300 Hz were detected significantly above the background at 6 cm (200 Hz, 6 cm: *t* ratio = 2.86, *p* = 0.019; 300 Hz, 6 cm: *t* ratio = 3.19, *p* = 0.011). While 600 Hz was only detectable above the background noise at a distance less than 1 cm (0.1 cm: *t* ratio = 2.74, *p* = 0.023), 900 Hz was not detected on the opposing leaf (0.1 cm: *t* ratio = 2.04, *p* = 0.072). While there was a small amount of signal loss from the shaker tip to the spot measured on the source leaf, there was significantly more signal loss to the closest distance measured on the target leaf (Fig. 5b).

## Discussion

Results from this study confirm a decrease in vibrational signal transmission intensity and loss of high-frequency components with increasing distances. The vibrations followed the expected patterns of greater propagation velocities for higher frequencies compared to lower frequencies. The effects of this dispersion pattern are discussed below. Furthermore, the lower frequency vibrational signals were found to bridge the gap from a leaf to the air to a non-connected leaf, thus extending the active space beyond the source plant for small vibrationally communicating insects.

### Frequency-dependent signal transmission

Theory of vibrations in beams suggests that signal transmission will occur in a frequency-dependent fashion. Indeed, this study determined that higher frequencies arrived significantly earlier than lower frequencies at distances as short as 50 cm on a grapevine. Measured phase velocity from this study fits well with established theory (Casas et al. 2007). Here, the phase velocity is related to the branch radius, *r* and wavelength, *λ* by

2 $$c = \frac{\pi r}{\lambda }\sqrt {\frac{E}{\rho }\left[ {1 + \frac{{\pi^{2} r^{2} }}{{3\lambda^{2} }}\left( {7 + 2\frac{E}{G} - 2\frac{G}{E}} \right)} \right]^{ - 1/2} ,}$$

where $G = \frac{E}{{2\left( {1 + \nu } \right)}}$ is the shear modulus, *E* the Young’s modulus, *ρ* density, and $\nu$ is Poisson’s ratio. *λ* is inversely proportional to frequency: $\lambda = \frac{c}{f}$. A simpler model valid in the low-frequency limit is given by

3 $$c = \frac{\pi r}{\lambda }\sqrt {\frac{E}{\rho }} .$$

The latter is referred to as the Timoshenko theory while the former is the Bernoulli−Euler theory. The two theories begin to diverge above about 1 kHz, but our study was not designed to test such high frequencies as they are biologically irrelevant for *H. vitripennis* communication. Parameters for the material properties of the branches were taken from Casas et al. (2007).

At 50 cm from the source, we found a 15 ms arrival-time difference between pure tones of 100 and 600 Hz, with the higher frequency arriving before the lower frequency. This difference in arrival time may affect the receiving animal’s perceived signal, as proposed in our model (Fig. 1b). A time difference of 15 ms or less has been shown to be detectable by the arthropod nervous system. For example, following a vibrational stimulus, the bimodal omega neuron of the field cricket, *Gryllus bimaculatus*, exhibited an inhibitory response with a 7 ms latency. When both vibrational and auditory stimuli were delivered to the cricket, a time difference of 15 ms between the signals resulted in the strongest inhibitory effect on the omega neuron (Wiese 1981). The latency for the vibrational interneuron response of the southern green stink bug, *Nezara viridula*, an insect more closely related to *H. vitripennis* than crickets, was 20−30 ms (Čokl and Amon 1980). Furthermore, studies of the nocturnal desert scorpion, *Paruroctonus mesaensis,* showed that it responded to time differences of 0.2 ms between vibrations delivered to different legs (Brownell and Farley 1979). Finally, insects, such as the lesser wax moth, *Achoria grisella*, use sequential signal analysis to orient and find their mates (Greenfield et al. 2002). Therefore, implications of our findings in light of these studies are that, in theory, arthropods may be able to use differences in the time of arrival of the harmonics to estimate the distance to and the direction of a duetting partner. Additional behavioral experiments are needed to confirm these hypotheses.

### Courtship signals

Because vibrational communication in plants may be noisy due to reflections and loss of signal intensity, identifying the distance to the signaler can be problematic, and may influence the communication patterns of the receiver. The female may be using the harmonic information from the male signal to determine whether he is orienting toward her and/or moving toward or away from her. One hypothesis is that if the female detects additional frequency components in each successive male signal, this would indicate a male’s approach and may trigger a reduction in her signaling activity to (a) conserve energy and/or (b) protect herself from nearby predators that may be eavesdropping. Conversely, if the female detects fewer harmonics in each successive male signal, she would continue to signal to provide a navigational beacon for the male. No discernible cue could be identified as the trigger to shift the female/male duetting pattern from a 1:1 calling ratio during the establishment of a duet (identification phase) to a 1:4 calling ratio during the searching phase (Nieri et al. 2017). Additional behavioral studies are needed to confirm this theory.

### Substrate and air-coupled communication

When considering the active space of vibrational signaling, the historic view was that vibrations extended as far as connecting substrates (Cocroft and Rodriguez 2005). However, recent work by Eriksson et al. (2011) demonstrated that the American grapevine leafhopper (*S. titanus*) can successfully signal across two non-connected leaves separated by a gap of up to 6 cm. Our efforts to replicate those results with frequency components of a *H. vitripennis* signal in the current study support their findings. We have shown that not all frequencies traverse the air gap with equal efficacy, as measured on the receiving leaf. Lower frequencies, e.g., 100 Hz, were detected on an opposing leaf at a distance of 10 cm—the furthest measured in this study. However, higher frequencies (200 and 300 Hz) could only be detected up to 6 cm; 600 Hz was only detected at a maximum distance of 0.1 cm and 900 Hz was undetectable above the background noise across gaps as small as 0.1 cm. Previous research identified similar leaf-to-air vibrational transmission, with much lower intensity signals than those studied here, especially in the high frequencies, detected in the air (Casas et al. 1998). In the case of the *H. vitripennis*, the first three harmonics (up to 300 Hz) are the most important for eliciting a response from duetting males (Mazzoni et al. 2017), and therefore, results from the current study suggest that animals calling on neighboring plants may be able to detect each other. In this way, call-fly behavior often seen in leafhoppers (Kuhelj et al. 2015) and identified in the *H. vitripennis* (SDG personal observation) may be initiated to increase the signaling space. In addition, a signal containing only the first three frequency components indicates that the receiver is far from the source. This in turn could act as the trigger to initiate duetting and search behavior, consistent with our model (Fig. 1b) that at long distances from the source, only a portion of the signal is present, indicating the animals are in the same general area and duetting and searching behaviors should occur. This is an example of how dispersion may be used as a localization mechanism. Furthermore, a recent study of the thornbug treehopper, *Umbonia crassicornis*, suggests adaptive decision making by males searching for females, based on the gradients of the signals received (Gibson and Cocroft 2018). Nevertheless, in her review, Mortimer (2017) was not able to identify any definitive examples of dispersion-based locating mechanisms used by animals.

### Vibration signal space

Taken together, the results from this study highlight the type of information that can enhance the active space of vibrationally signaling animals. While with increasing distances the signal amplitude clearly decreases (Fig. 4d), there is possibly added information transmitted based on the timing of arrival of signal components of different frequencies. Arthropods may be able to use this information as an additional cue to estimate the distance to their signaling partner. Moreover, the vibrational signals are able to propagate beyond their source plant by the leaves acting as signal emitters and receivers. In this case, transmission of the lower frequency components is favored, which again, could indicate the presence of a distant conspecific. Now that vibrational studies using portable laser Doppler vibrometries of both invertebrates and vertebrates in their natural habitat are becoming more widespread, detailed measurements of the active space of vibrational signals are more tractable.

## Electronic supplementary material

Below is the link to the electronic supplementary material.

Supplementary material 1: Supplemental Video 1. A sample recording of the vine movement at 100 Hz from the Polytec software showing the z-axis of movement of the signal traveling along the vine. Green and red dots are the extreme in and out of plane maximum and minimum values. (MOV 19,240 kb)

Supplementary material 2: Supplemental Video 2–6. A cartoon showing the wave front over time. The colors represent distance from the source in meters with red at the mini-shaker to blue furthest away. Videos are for 100 Hz, 200 Hz, 300 Hz, 600 Hz, and 900 Hz. (MOV 1062 kb)

Supplementary material 3 (MOV 1003 kb)

Supplementary material 4 (MOV 910 kb)

Supplementary material 5 (MOV 641 kb)

Supplementary material 6 (MOV 1503 kb)

## References

[CR1] Barth FG, Lehrer M (1997). Vibratory communication in spiders: adaptation and compromise at many levels. Orientation and communication in arthropods.

[CR2] Barth FG, Hoy RR, Popper AN, Fay RR (1998). The Vibrational Sense of Spiders. Comparative hearing: insects. Springer handbook of auditory research.

[CR3] Brenowitz EA (1982). The active space of red-winged blackbird song. J Comp Physiol A.

[CR4] Brownell P, Farley RD (1979). Orientation to vibrations in sand by the nocturnal scorpion *Paruroctonus mesaensis*: mechanism of target localization. J Comp Physiol A.

[CR5] Casas J, Bacher S, Tautz J, Meyhofer R, Pierre D (1998). Leaf vibrations and air movements in a leafminer−parasitoid system. Bio control.

[CR6] Casas J, Magal C, Sueur J (2007). Dispersive and non-dispersive waves through plants: implications for arthropod vibratory communication. Proc R Soc B.

[CR7] Cocroft RB, Rodriguez RL (2005). The behavioral ecology of insect vibrational communication. BioSci.

[CR8] Čokl A, Amon T (1980). Vibratory interneurons in the central nervous system of *Nezara viridula* L. (Pentatomidae, Heteroptera). J Comp Physiol A.

[CR9] Čokl A, Virant-Doberlet M (2003). Communication with substrate-borne signals in small plant-dwelling insects. Ann Rev Entomol.

[CR10] Čokl A, Laumann RA, Kosi AZ, Blassioli-Moraes MC, Virant-Doberlet M, Borges M (2015). Interference of overlapping insect vibratory communication signals: an *Eushistus heros* model. PLoS ONE.

[CR11] Egnor SER, Wickelgren JG, Hauser MD (2007). Tracking silence: adjusting vocal production to avoid acoustic interference. J Comp Physiol A.

[CR12] Eriksson A, Anfora G, Lucchi A, Virant-Doberlet M, Mazzoni V (2011). Inter-plant vibrational communication in a leafhopper insect. PLoS ONE.

[CR13] Gibson JS, Cocroft RB (2018). Vibration-guided mate searching in treehoppers: directional accuracy and sampling strategies in a complex sensory environment. J Exp Biol.

[CR14] Gordon SD, Uetz GW (2011). Multimodal communication of wolf spiders on different substrates: evidence for behavioural flexibility. Anim Behav.

[CR15] Gordon SD, Sandoval N, Mazzoni V, Krugner R (2017). Mating interference of glassy-winged sharpshooters, *Homalodisca vitripennis*. Entomol Exp Appl.

[CR16] Greenfield MD, Tourtellot MK, Tillber C, Bell WJ, Prins N (2002). Acoustic orientation via sequential comparison in an ultrasonic moth. Naturwissenschaften.

[CR17] Halfwerk W, Slabbekoorn H (2009). A behavioural mechanism explaining noise-dependent frequency use in urban birdsong. Anim Behav.

[CR18] Krugner R, Gordon SD (2018). Mating disruption of *Homalodisca vitripennis* (Germar) (Hemiptera: Cicadellidae) by playback of vibrational signals in vineyard trellis. Pest Man Sci.

[CR19] Kuhelj A, DeGroot M, Pajk F, Simčič T, Virant-Doberlet M (2015). Energetic cost of vibrational signalling in a leafhopper. Behav Ecol Sociobiol.

[CR20] Lardner B, bin Lakim M (2002). Tree-hole frogs exploit resonance effects. Nature.

[CR21] Lopez PT, Narins PM, Lewis ER, Moore SW (1988). Acoustically induced call modification in the white-lipped frog *Leptodactylus albilabris*. Anim Behav.

[CR22] Markl H, Huber F, Markl H (1983). Vibrational communication. Neuroethology and behavioral physiology.

[CR23] Mazzoni V, Eriksson A, Anfora G, Lucchi A, Virant-Doberlet M, Cocroft RB, Gogala M, Hill PSM, Wessel A (2014). Active space and the role of amplitude in plant-borne vibrational communication. Studying vibrational communication.

[CR24] Mazzoni V, Gordon SD, Nieri R, Krugner R (2017). Design of a candidate vibrational signal for mating disruption against the glassy-winged sharpshooter, *Homalodisca vitripennis*. Pest Man Sci.

[CR25] Michelsen A, Fink F, Gogala M, Traue D (1982). Plants as transmission channels for insect vibrational songs. Behav Ecol Sociobiol.

[CR26] Miles RN (2016). An analytical model for the propagation of bending waves on a plant stem due to vibration of an attached insect. Heliyon.

[CR27] Mortimer B (2017). Biotremology: do physical constraints limit the propagation of vibrational information?. Anim Behav.

[CR28] Narins PM, Hödl W, Grabul DS (2003). Bimodal signal requisite for agonistic behavior in a dart-poison frog *Epipedobates femoralis*. Proc Nat Acad Sci.

[CR29] Narins PM, Grabul DS, Soma K, Gaucher P, Hödl W (2005). Cross-modal integration in a dart-poison frog. Proc Nat Acad Sci.

[CR30] Nieri R, Mazzoni V, Gordon SD, Krugner R (2017). Mating behavior and vibrational mimicry in the glassy-winged sharpshooter, *Homalodisca vitripennis*. J Pest Sci.

[CR31] Partan S, Marler P (2005). Issues in the classification of multimodal communication signals. Am Nat.

[CR32] Polajnar J, Svenšek D, Čokl A (2012). Resonance in herbaceous plant stems as a factor in vibrational communication of pentatomid bugs (Heteroptera: Pentatomidae). J R Soc Inter.

[CR33] Rovner JS, Barth FG (1981). Vibratory communication through living plants by a tropical wandering spider. Science.

[CR34] Schneider CA, Rasband WS, Eliceiri KW (2012). NIH Image to ImageJ: 25 years of image analysis. Nat Methods.

[CR35] Wiese K (1981). Influence of vibration on cricket hearing: interaction of low frequency vibration and acoustic stimuli in the omega neuron. J Comp Physiol.

[CR36] Wells KD, Schwartz JJ (1984). Vocal communication in a neotropical treefrog, *Hyla ebraccata*: advertisement calls. Anim Behav.

