# Correction to: Transmission of the frequency components of the vibrational signal of the glassy-winged sharpshooter, *Homalodisca vitripennis,* within and between grapevines

**DOI:** 10.1007/s00359-019-01368-8

**Published:** 2019-09-17

**Authors:** Shira D. Gordon, Benjamin Tiller, James F. C. Windmill, Rodrigo Krugner, Peter M. Narins

**Affiliations:** 1 Agricultural Research Service, San Joaquin Valley Agricultural Sciences Center, United States Department of Agriculture, 9611 S Riverbend Ave, Parlier, CA 93648 USA; 2 Department of Electronic and Electrical Engineering, Centre for Ultrasonic Engineering, University of Strathclyde, Glasgow, Scotland G1 1XW UK; 3 Department of Integrative Biology and Physiology, UCLA, Los Angeles, CA 90095 USA

## Correction to: Journal of Comparative Physiology A 10.1007/s00359-019-01366-w

Unfortunately, Fig. 3 was incorrectly published in the original publication. The correct version of Fig. 3 is updated here.

**Fig. 3:**
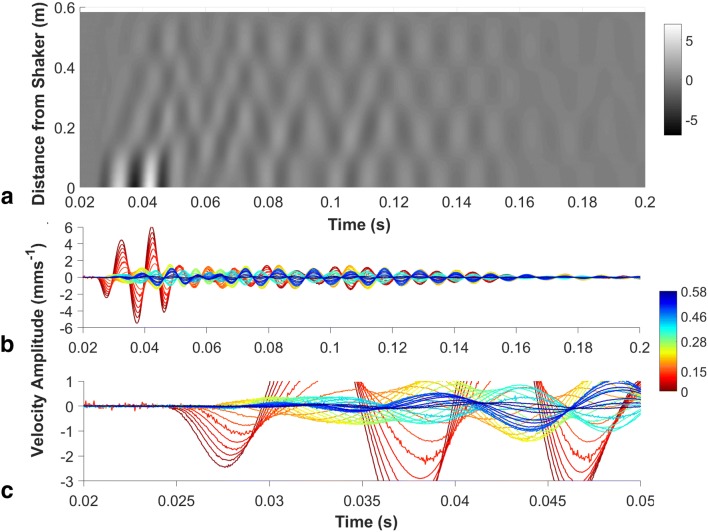
Sample data of signal transmission on one vine at 100 Hz in **a** matrix plot of time and distance (gray scale is velocity amplitude mm s^−1^); **b** time vs. velocity amplitude at different distances from the source (colors are the distances from the source in meters, with red at the mini-shaker to blue furthest away); **c** magnified view of **b** showing details of the low-amplitude traces. Sample data from different frequencies can also be seen as a video representation for each frequency in the supplemental materials (suppl. video 1−6)

